# Social Media in Health Science Education: An International Survey

**DOI:** 10.2196/mededu.6304

**Published:** 2017-01-04

**Authors:** Elizabeth O'Sullivan, Emily Cutts, Sushma Kavikondala, Alejandra Salcedo, Karan D'Souza, Martin Hernandez-Torre, Claire Anderson, Agnes Tiwari, Kendall Ho, Jason Last

**Affiliations:** ^1^ School of Medicine University College Dublin Dublin Ireland; ^2^ School of Pharmacy University of Nottingham Nottingham United Kingdom; ^3^ School of Nursing University of Hong Kong Hong Kong China; ^4^ Escuela Nacional de Medicina Tecnológico de Monterrey Monterrey Mexico; ^5^ Faculty of Medicine University of British Columbia Vancouver, BC Canada

**Keywords:** health education, health surveys, interdisciplinary studies, learning, professionalism, self report, social media, students, surveys and questionnaires, universities

## Abstract

**Background:**

Social media is an asset that higher education students can use for an array of purposes. Studies have shown the merits of social media use in educational settings; however, its adoption in health science education has been slow, and the contributing reasons remain unclear.

**Objective:**

This multidisciplinary study aimed to examine health science students’ opinions on the use of social media in health science education and identify factors that may discourage its use.

**Methods:**

Data were collected from the Universitas 21 “Use of social media in health education” survey, distributed electronically among the health science staff and students from 8 universities in 7 countries. The 1640 student respondents were grouped as users or nonusers based on their reported frequency of social media use in their education.

**Results:**

Of the 1640 respondents, 1343 (81.89%) use social media in their education. Only 462 of the 1320 (35.00%) respondents have received specific social media training, and of those who have not, the majority (64.9%, 608/936) would like the opportunity. Users and nonusers reported the same 3 factors as the top barriers to their use of social media: uncertainty on policies, concerns about professionalism, and lack of support from the department. Nonusers reported all the barriers more frequently and almost half of nonusers reported not knowing how to incorporate social media into their learning. Among users, more than one fifth (20.5%, 50/243) of students who use social media “almost always” reported sharing clinical images without explicit permission.

**Conclusions:**

Our global, interdisciplinary study demonstrates that a significant number of students across all health science disciplines self-reported sharing clinical images inappropriately, and thus request the need for policies and training specific to social media use in health science education.

## Introduction

Social media facilitates information sharing, including usergenerated content, and has transformed the way we communicate. As of 2010, The Millennial Generation, individuals born between 1980 and 2000, comprised the major users of social media, with approximately 75% having a personal social networking page (eg, Facebook profile) and 61% perceiving sharing of personal data and images through social media as positive [1].

Health care professionals and health science students use social media as much as the general population [2] with approximately 90% of practicing doctors, nursing staff, and allied health care professionals having Facebook pages for personal or professional use [3]. Social media has implications in health science education due to patient and provider confidentiality; however, till date there is little instruction in the mainstream health science education to help students securely and appropriately engage with digital media [4,5], and wherever these guidelines are available, they have been mostly created by practicing health care professionals and academicians without the input of students and often lack definitions of professionalism as applied to online presence [6]. In recent years, a few studies incorporating the views of the broader health care provider community have come up with guidelines and frameworks that could help health science students and health care professionals to better embrace the positive aspects of social media in health care [4,7], although much needs to be done by universities and professional bodies in incorporating and providing these guidelines to students and professionals.

A recent survey among medical students showed that there is little consensus on what constitutes unprofessional behavior beyond the US Health Insurance Portability Act violations and students have felt that posting inappropriate material on personal social media sites was “unavoidable” [8,9]. Furthermore, studies have reported that students are unaware of ethical concerns posed by social media usage [10]; and even if students are aware of the importance of online professionalism, they do not feel it is relevant to them until they graduate and have an actual online professional identity [11]. Studies have also reported that students do not want or need formal policies for posting content online [9] and in fact, considered any enquiries into their social media use as “intrusive” and believed social media use to be too personal a topic for discussion [12]. Health science students struggle with the concepts associated with professionalism [13,14], and often fail to recognize the effect of their social media activities on future professional goals. In an age of growing social media influence and an increase in the perceived distrust of health care professionals by the public [15], it is important for schools to use an evidence-based approach to policy creation and to involve students in the process of the creation of these policies.

Although focus groups, surveys, and reviews of the literature have gathered usage information and perspectives from medical students and doctors [8,16,17], including a study that gathered information on health science students’ media preferences and how often they use social media sites, and evaluated their responses to advertisements [18], no study to our knowledge, has examined the user profiles, attitudes, and perspectives of students from multiple disciplines and multiple cultures on the use of social media in health science education. Understanding the demographics and perceptions of students in different health science disciplines may be imperative to developing better student guidelines.

The purpose of this study was to examine the use of social media by students in health science education as well as the barriers to its use. By doing so, we can identify what could promote social media’s use as an educational tool among health science students as well as how to improve its appropriate use.

## Methods

The Universitas 21 Health Science Group (U21HSG) is a group of research-intensive universities committed to working together and pooling resources to conduct research in health science education. U21HSG conducted this large-scale, international study that involved 8 universities, which explored the user demographics, perceptions, and usage behaviors of dentistry, medical, nursing, pharmacy, physiotherapy, public health, and other allied health care students to social media use.

U21HSG developed an extensive survey to explore how social media is being used in health science education as well as educators’ and students’ opinions on it. The survey was trialled with students and faculty members within U21HSG first. Based on the feedback, we modified the survey and then distributed it subsequently in a more widespread approach. The results from the original trial were not included in our final analysis. The survey was first distributed among the members of the group as a trial. Feedback was received from the group, and the survey was modified accordingly before distribution. Prior to the distribution of the survey, an ethical approval was sought and granted from all 8 institutions. The Web-based survey was hosted using the FluidSurveys (SurveyMonkey) platform and was distributed among health science educators and students in the following 8 universities: Fudan University (China), Tecnologico de Monterrey (Mexico), University College Dublin (Ireland), University of British Columbia (Canada), University of Nottingham (United Kingdom), University of Birmingham (United Kingdom), Hong Kong University (Hong Kong), and the University of Melbourne (Australia). Responses to the survey were anonymous and were received between April and October 2014.

For the purpose of the survey, social media was described to participants as “a rapidly developing group of powerful and ubiquitous technologies and set of sociotechnical approaches for people to connect, support, and learn from each other. In other words, any online platform in which people communicate with each other, for example, Twitter, Facebook, YouTube, wikis, blogs, and so on.”

Excluding educator responses left us with 2059 respondents, of which 419 either did not identify themselves as students or did not complete more than one question, and thus were excluded from the analysis. A total number of 1640 student responses were received and included in this analysis. The significance threshold set was 0.05 (*P*<.05 is significant).

Students who reported using social media in their education “never” or “rarely” were categorized as “nonusers,” whereas those who reported using social media “sometimes,” “often,” or “almost always” were categorized as “users.” Respondents were divided into these 2 groups to see if users and nonusers use social media differently or view social media use in health science education differently.

## Results

Of the 1640 student respondents, 1343 (81.89%) were users and 297 (18.11%) were nonusers. Usage across the health science disciplines ranged from 63% in pharmacy to 91% in physiotherapy with the mean usage of 80% across all disciplines.

Table 1 exhibits the demographics of the respondents and the relationship between the demographic factors and the usage. There was a statistically significant difference between the mean age of users and nonusers (*P*=.003); however, there was no significant difference in the usage of social media between men and women (*P*>.99).

**Table 1:** Demographic characteristics of users and nonusers of social media.

Demographic characteristics		Users	Nonusers	Total	*P* value
Age in years, mean (SD)		n=1351 23.1 (4.74)	n=297 24.05 (5.68)	n=1648 23.25 (4.96)	.003
**Gender, n (%)**		n=1342	n=297	n=1639	>.99
	Male	444 (81.9)	98 (18.1)	542 (33.07)	
	Female	897 (81.84)	199 (18.16)	1096 (66.87)	
	Nonbinary	1 (100)	0 (0)	1 (<0.1)	
**University affiliation, n (%)**		n=1505	n=296	n=1801	.001
	Fudan University	55 (78)	16 (23)	71 (3.94)	
	Tecnologico de Monterrey	491 (89.9)	55 (10.1)	546 (30.31)	
	University College Dublin	336 (82.2)	73 (17.8)	409 (22.71)	
	University of Birmingham	58 (78)	16 (22)	74 (4.11)	
	University of British Columbia	52 (72)	20 (28)	72 (3.99)	
	University of Hong Kong	83 (94)	5 (6)	88 (4.88)	
	University of Melbourne	100 (86.9)	15 (13.1)	115 (6.38)	
	University of Nottingham	330 (77.5)	96 (22.5)	426 (23.65)	

Among both users and nonusers, uncertainty on policies (51%, 68%, respectively), concerns about professionalism (46%, 57%), and lack of support from the department (39%, 57%) were the 3 biggest barriers to social media use. However, a much larger proportion of nonusers (47.0%, 119/253) did not understand how to incorporate it into their learning, compared with users (11.99%, 140/1167). Only 6.94% (80/1152) of the users failed to see the value of social media in education, compared with 29.8% (74/248) of nonusers (*P*<.001). Every barrier was more often reported by nonusers than users. The largest barrier among both groups was uncertainty on policy, which varied from institution to institution and ranged from 34% to 80%. The mean for all 8 global universities was 60%.

**Figure 1:**
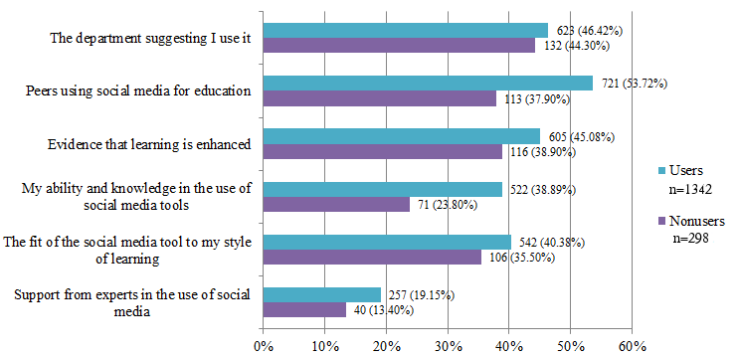
Factors influencing social media use by health science students.

Factors that would encourage students to use social media in their education are shown in Figure 1. Departments had a big influence on students’ social media use, as did peers. Evidence that social media use will enhance their learning would encourage users and nonusers alike to use social media.

Table 2 demonstrates that 858 of 1320 (65.00%) respondents did not receive training in social media policies and guidelines and that the majority of those who did not receive training would like to. The impact of social media training on students’ confidence in using social media is also examined in Table 2.

**Table 2:** Health science students’ social media training.

Social media training		Users	Nonusers	Total
**Training received on social media policy or guidelines from the faculty, n (%)**		n=1091	n=229	n=1320
	Yes	393 (36.02)	69 (30.1)	462 (35.00)
	No	698 (63.98)	160 (69.9)	858 (65.00)
**Would like to receive training on social media policy or guidelines, n (%)**		n=762	n=174	n=936
	Yes	511 (67.1)	94 (54.0)	608 (64.9)
	No	251 (32.9)	82 (46.0)	328 (35.1)
**Did training increase confidence?**		n=418	n=75	n=493
	It increased confidence	250 (60.1)	28 (37)	278 (56.4)
	It didn’t affect confidence	168 (39.9)	47 (63)	215 (43.6)

**Figure 2:**
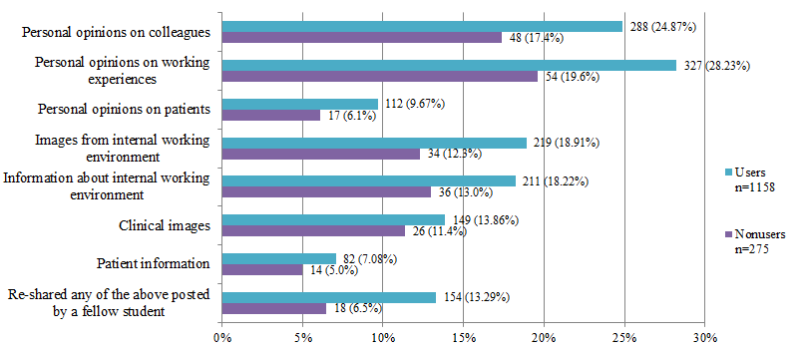
Inappropriate sharing of information by users and nonusers on social media.

Figure 2 shows the rates of sharing of different items among users and nonusers without explicit permission. Nonusers had lower rates of inappropriate sharing in all categories than their user counterparts. Both groups most often shared opinions on work experiences. More than 10% of both users and nonusers have shared clinical images without explicit permission.

Social media nonusers had fewer reported breaches of confidentiality. Table 3 represents inappropriate sharing among users and nonusers and is broken down by their frequency of use. Those who used social media the most had the highest rate of inappropriate sharing of each category; however, nonusers did not always have the lowest rate.

Of the 174 respondents who had shared clinical images without permission, 50 (28.7%) either did not use security settings or did not know what their security settings were.

**Table 3:** Frequency of social media use and inappropriate sharing by health science students.

Information shared on social media	Nonusers n (%)	Sometimes n (%)	Often n (%)	Almost always n (%)
Patient information	82 (6)	29 (8)	25 (5)	29 (12)
Clinical images	26 (12)	44 (12)	55 (11)	50 (21)
Information about internal working environment	36 (16)	53 (15)	94 (19)	66 (28)
Images from working environment	34 (15)	54 (15)	89 (18)	78 (33)
Personal opinions on patients	17 (8)	39 (11)	42 (9)	31 (13)
Personal opinions on working experiences	54 (25)	85 (24)	154 (32)	92 (38)
Personal opinions on colleagues	49 (22)	75 (22)	129 (26)	86 (36)
Reshared any of the above posted by a fellow student	18 (8)	43 (12%)	55 (12)	56 (24)

## Discussion

### Principal Findings

As expected, most students (81.89% in total) from all health science disciplines were already using social media in their education. The biggest barriers to social media use among both users and nonusers were uncertainty on policies, concerns about professionalism, and lack of support from the department. All barriers were reported more frequently by nonusers than users. Not understanding how to incorporate social media into their learning is a barrier to almost half of the nonusers. Even a small portion of users reported not being sure of how to incorporate social media into their education. Having identified the 3 biggest barriers to health science students, institutions can understand the worries of their students and make guidelines and courses to help them become more comfortable with social media use.

Lack of support from the departments was one of the biggest barriers to social media use among users and nonusers alike and both groups identified that departments suggesting its use would influence their use of social media in their education. A similar study found that faculty reluctance was a barrier to social networking sites being used in third-level teaching [13]. Departments and faculty have a large influence on student’s use of social media in their education. Educational institutions need to identify ways to increase the pedagogical value of social media to encourage usage while establishing clear guidelines to support positive and healthy use of social media.

The fact that most students who did not receive social media use training and reported that they would want it in the future is a positive sign. Kind et al reported that students did not want or need formal policies for posting content online; however, our findings suggest the opposite that students want formal policies for posting content on social media [4]. Perhaps, student needs have changed with the rise in social media use since the paper was published in 2010, and we only expect this trend to continue as social media continues to grow and integrate as a learning tool.

More than half of the users who received training on social media policy reported that it increased their confidence in using social media for educational purposes, implying that this training was beneficial for these students. Perhaps there is room for more research among those who did not find the training beneficial, so that training can be improved based on the feedback of students. It would also be important to provide nonusers with ways to incorporate social media into education to increase their usage. Having frequent users or faculty members showing good practices of social media use in education would be a good way to support nonusers for social media uptake.

Health science students have access to personal information about patients that must be kept confidential. This study shows that students across these 8 institutions distributed internationally share an alarming amount of inappropriate clinical information. The students who use social media the most reported a worrying amount of inappropriate sharing of clinical images. This figure (20.5%) substantiates one of the biggest concerns of social media use in health science education— confidentiality. Our findings suggest that appropriate training, policies, and guidelines be put in place to curb this. Students and faculty working together to ensure good practice and respect for patient privacy and confidentiality will play an important role in reducing the rate of inappropriate posting. Once social media is introduced with due care, it will be supported even by skeptics.

### Conclusions

Although social media is being used for learning purposes by most health science students across the globe, many do so without appropriate training. Also, a high rate of inappropriate posting of content without explicit permission was self-reported, thereby jeopardizing patient confidentiality and the student-patient relationship. Meanwhile, students are receptive to training in social media use, and having faculty’s support can facilitate increase in social media usage for enhancement of education. Faculty clearly has an important role to play in ensuring social media’s safe use by students. Ideally, staff should integrate social media education and policies into curricula to ensure that students are making the most of these digital assets and are doing so with the least possible risk.

Our findings suggest that training programs to engage students in social media policy with clear benefits of social media in health science be made and implemented in institutions around the world. The training should include guidance on how and when to report a breach of the policy, along with consequences of breaking the rules. By implementing a training program, it is envisaged more students would not only be aware of and adhere to the policy but also know how social media can be used in an effective and safe manner for the ultimate benefit of patients.

## Abbreviations

U21HSG: Universitas 21 Health Science Group
